# The integrated role of multiple healthy weight behaviours on overweight and obesity among adolescents: a cross-sectional study

**DOI:** 10.1186/s12889-019-7007-7

**Published:** 2019-08-22

**Authors:** Sandya Menon, Anne Philipneri, Sujitha Ratnasingham, Heather Manson

**Affiliations:** 1 Public Health Ontario, 480 University Avenue #300, Toronto, Ontario M5G 1V2 Canada; 2 Institute for Clinical Evaluative Sciences, 2075 Bayview Ave, Toronto, ON M4N 3M5 Canada; 3 Dalla Lana School of Public Health, University of Toronto, 480 University Avenue #300, Toronto, Ontario M5G 1V2 Canada

**Keywords:** Adolescents, Obesity, Physical Activity, Screen time, Fruit and Vegetable Consumption, Sleep, Recommendations

## Abstract

**Background:**

This study contributes to the limited number of studies that have explored the impact of not meeting the recommendations for moderate-to-vigorous physical activity, screen time, fruit and vegetable consumption and sleep on overweight and obesity among adolescents.

**Methods:**

A cross-sectional study of data from the 2015 *Ontario Student Drug Use and Health Survey* (OSDUHS), a provincially representative survey of students in publically funded schools in Ontario, Canada, was conducted. This study included self-reported data from students aged 11–17 years (*n* = 9866). The main outcome variable was overweight or obesity, classified using WHO BMI cut-points. Four independent variables for healthy weight behaviours were examined: (1) moderate-to-vigorous physical activity (MVPA) (≥ 60 mins vs. < 60 mins everyday over the last seven days); (2) screen time (< 2 h daily vs. ≥ 2 h daily); (3) fruit and vegetable consumption (≥ 5 times/day vs. < 5 times/day); (4) sleep (adequate based on guidelines vs. inadequate). Covariates included sex, age, Subjective Social Status (SSS), parental education and ethnicity. Binomial and multinomial logistic regression models were fitted to determine whether not meeting the recommendations for healthy weight behaviours was associated with overweight or obesity status.

**Results:**

Only 2% of students in Ontario met the recommendations for all four healthy weight behaviours and 33% of students did not meet any of the four recommendations. In both the binomial and multinomial models, not meeting the recommendations for MVPA was the only significant healthy weight behaviour associated with both overweight and obesity (AOR: 1.29, 95% CI: 1.03–1.62), and solely obesity (AOR: 1.45, 95% CI: 1.05–1.99). Males, students with lower SSS ratings, and students with parents with an education of ‘High School’ or less were also at significantly greater odds of being obese.

**Conclusion:**

Findings from this study show that inadequate levels of MVPA is a critical behavioural predictor of obesity status in adolescents between the ages of 11–17 years, after controlling for differences in screen time, fruit and vegetable consumption, sleep, and demographics. Findings from this study could have implications toward policies and programs targeted at reducing obesity, and increasing the physical activity rates of adolescents.

## Background

The issue of childhood and adolescent overweight and obesity has consistently been identified as a leading public and global health priority [1, 2]. The ensuing health risks and chronic conditions that can develop and persist into adulthood have been well-documented, which include but are not limited to, cardiovascular disease, diabetes and various cancers [1]. As of 2016, the global prevalence of overweight and obesity was reported to be over 18% of all 5–19 year olds, a 14% increase since 1975 [1]. Within Canada alone, the prevalence of obesity among children and adolescents between the ages of 5–17 years has more than doubled in the last three decades, with nearly one-third of children and adolescents being reported as being either overweight or obese as of 2013 [3, 4]. In consideration of these prominent rates, there is a need to better understand the modifiable risk factors that contribute to increased weight status, in order to better inform policies and interventions aiming to reduce the burden of overweight and obesity among this demographic.

Engagement in select lifestyle behaviours has been shown to impact the likelihood of overweight and obesity in school-aged children and adolescents. Previous studies have shown that moderate-to-vigorous-physical activity (MVPA), screen time and sleep are independently associated with adiposity across varying study designs, including possible dose-response relationships between these modifiable behavioural determinants and adiposity [5–7]. Increased MVPA and longer sleep have been associated with lower adiposity indicators among adolescents, while excessive screen time engagement has shown an increased risk for obesity [5–7].

Fruit and vegetable consumption has also been described as a modifiable behavioural determinant of overweight and obesity, though shown associations in previous studies has been weak and inconsistent among adolescents [8]. However, as fruit and vegetable consumption is commonly considered an indicator for overall diet quality, the implications of dietary behaviours with movement behaviours on weight status should be measured [8, 9]. Further examination of the relationships between lifestyle behaviours and body weight could help to better elucidate the strength and consistency of these associations on the weight status of adolescents.

While the isolated relationships between individual healthy weight behaviours and adolescent weight status have been extensively investigated, exploration of the impact of concurrent engagement in more than two healthy weight behaviours is generally under-studied. Within a Canadian context, some studies have examined three of the four aforementioned behaviours (MVPA, screen time and sleep), in light of the release of the *24-h Movement Guidelines for Canadian Children and Youth: An Integration of Physical Activity, Sedentary Behaviour and Sleep,* by the Canadian Society for Exercise Physiology [10–12]. Other studies that have examined the associations between all four behaviours (MVPA, screen time, diet and sleep) cannot be applied to a North American context, as their study population would not be representative of North American adolescents [13–15]. Current global and Canadian rates of childhood and adolescent overweight and obesity warrant the need for further research that addresses the influence of lifestyle behaviours on BMI status among this age demographic using an integrated approach.

Through use of a provincially-representative dataset, up-to-date evidence-based national recommendations, and exploration within a Canadian context of four healthy lifestyle behaviours, this study aims to contribute to the limited body of evidence examining the prevalence and integrated role of healthy weight behaviours on overweight and obesity status among adolescents.

## Methods

### Data

Data for this study were obtained from the 2015 *Ontario Student Drug Use and Health Survey* (OSDUHS) (*n* = 10,426). The OSDUHS is a repeat-cross-sectional provincial survey that collects information on smoking, alcohol, drug use, and health-related behaviours. Participants are students between grades 7–12 attending publically funded English and French speaking schools in Ontario, Canada. The survey is estimated to represent 92% [16] of all grade 7–12 students in Ontario and excludes those in private schools, home-schools, institutions for correctional or health reasons, First Nations reserve schools, military bases, and geographically inaccessible areas. Further details regarding the OSDUHS can be found elsewhere [16].

This study restricted the sample only to students aged 11–17 years in grades 7–12, to adhere to the age cut-offs used by the nationally established guidelines for MVPA, screen time and sleep [10]. Thus, students aged 18–20 years (*n* = 560) were excluded from this study.

### Independent variables

Student healthy weight behaviours were measured using a self-report questionnaire. Moderate-to-vigorous physical activity (MVPA) was measured using the following question: “On how many of the last 7 days were you physically active for a total of at least 60 minutes on each day? Please add up all the time you spent on any kind of physical activity that increased your heart rate and made you breathe hard some of the time (Some examples are brisk walking, running, rollerblading, biking, dancing, skateboarding, swimming, soccer, basketball, football.) Please include both school and non-school activities.” Response options ranged from 0 to 7 days. As according to the 2016 *Canadian 24-h Movement Guidelines for Children and Youth* [10], students who did not report getting at least 60 min of MVPA each day over the last 7 days were categorized as not having met the guidelines. This questionnaire item has been used in CDC’s Youth Risk Behaviour Survey (YRBS) and WHO’s Health Behaviour in School-aged Children (HBSC) survey previously, and has been shown to capture responses that were valid and reliable [17, 18]. For Screen Time, participants were asked “In the last 7 days, about how many hours did you spend: watching TV/movies, playing video/computer games, on a computer/tablet chatting, emailing or surfing the Internet in your free time?” Response options ranged from none to 7 h or more a day. Students who reported spending more than 2 h daily in front of a screen were categorized as not having met the guidelines [10]. Students who responded as “Not Sure” were excluded from the analysis. This questionnaire item has also been used in WHO’s Health Behaviour in School-aged Children (HBSC) survey previously, and has been shown to capture responses that were valid and reliable [18]. For Fruit and Vegetable Consumption, participants were asked “On an average day, how many times do you eat fruits and vegetables?” with the following response options: [1] 0 times a day; [2] 1 time a day; [3] 2 times a day; [4] 3 times a day; [5] 4 times a day; [6] 5 times a day; [7] 6 or more times a day. This study classified students who did not consume fruits and vegetables at least five times a day as not having met the Association for Public Health Epidemiologists of Ontario (APHEO) Indicator for Vegetable Consumption, which is consistent with cut-offs used in other research [19–21]. For Sleep duration, students were asked “On an average night, how many hours of sleep do you get?” Response options ranged from 4 h or less, to 10 h or more. Based on the age-specific cut-offs, students between the ages of 11–13 years who did not report getting 9–11 h of nightly sleep, and students between 14 and 17 years of age who did not report getting 8–10 h of nightly sleep were categorized as not having met the guidelines [10]. This questionnaire item has been used in CDC’s Youth Risk Behaviour Survey (YRBS) and WHO’s Health Behaviour in School-aged Children (HBSC) survey previously, and has been shown to capture responses that were valid and reliable [17, 18].

### Dependent variable

Students’ height and weight were self-reported from a list of given height and weight measurements, provided in inches and centimetres, and pounds and kilograms, respectively. From these measurements, BMI z-scores were derived using the World Health Organization (WHO) growth reference standard [22]. Using the WHO classification, four groups were classified: overweight (z-score > + 1 Standard Deviation (SD)), obese (> + 2SD), normal (between + 1 and − 2 SD) and thinness (> − 2SD). In the initial analysis, these four groups were combined into two groups: (1) overweight and obese, and (2) not overweight and obese. The initial analysis was followed by a secondary analysis, consisting of three groups: (1) obese (2) overweight and (3) not overweight and obese.

### Demographic variables

Age was captured as a continuous measure. For student ethnicity, participants were asked “Which of the following best describes your background (You may choose more than one background),” and provided with 13 ethnicity response options. The following ethnicity groups were created, as according to previously established groupings using OSDUHS data: [23] White, Black, Asian (South Asian, Southeast Asian, Chinese, Japanese, Korean and Filipino) and Other (West Asian or Arab, Aboriginal/First Nations, Latin American, Multi-ethnic). Student-reported paternal and maternal education attainment were combined into a single composite variable with the following groups: (1) University/College Graduate; (2) Some Post-Secondary Education, (3) High School Graduate or Less, where data for the parent with the highest education level was used, as done previously [23]. Subjective Social Status (Scale from 1 to 10), measured using the MacArthur Scale of Subjective Social Status (SSS), was also captured as a continuous measure. Parental education attainment and SSS were included simultaneously in models based on previous literature suggesting that each variable measures a different component of social status [23, 24].

### Statistical analyses

Descriptive statistics were computed for all independent, dependent and socio-demographic variables to observe the distribution by BMI status. Associations between the categorical variables and BMI status were examined using second-order design-adjusted Rao-Scott Modified Chi-Squared tests to account for the complex survey design [14]. Scaled Rectangle Diagrams were created in R to visually describe the prevalence of students meeting recommendations for multiple healthy weight behaviours [25].

Relationships between the four healthy weight behaviours and BMI status in students were explored using multivariable binary and multinomial logistic regression models. The binary logistic regression outcome variable was categorized by students being overweight and obese, and students not being overweight or obese. The multinomial logistic regression outcome variable consisted of three outcome levels: (1) obese, (2) overweight, and (3) not overweight or obese. Regression models were built beginning with the four healthy weight behaviours, followed by adjustment for co-variates. All analyses accounted for the complex sampling strategy of OSDUHS and applied the weight, strata, and cluster variables to obtain estimates relative to the target population. Reported values were verified to be meeting the release criteria for OSDUHS (coefficient of variation < 33.3). Statistical significance was examined at α = 0.05 level, unless otherwise noted. All analyses were conducted on SAS V. 9.3 [26].

## Results

The study sample consisted of 9866 students. The majority of students were in high school, with the mean age being 14.9 years. Male students (49%) represented a slightly lower percentage of the overall population. Students who reported being White represented the largest ethnic group (59%). As reported by students, the majority of parents were University or College graduates (81%) (Table 1).

**Table 1:** Socio-demographic characteristics of OSDUHS students by BMI Status, 2015 (*n* = 9866)

Characteristics	Overall		Overweight/Obese		Not Overweight/Obese	
	%	(95% CI)	%	(95% CI)	%	(95% CI)
Sex						
Male	48.8	(46.2–51.5)	33.4**	(30.8–36.1)	66.6	(63.9–69.2)
Female	51.2	(48.5–53.8)	25.3	(22.8–27.7)	74.8	(72.3–77.2)
Age (years)						
11–13 years old	27.0	(24.7–29.3)	30.0	(26.2–33.9)	70.0	(66.1–73.8)
14–17 years old	73.0	(70.7–75.3)	29.3	(27.2–31.3)	70.8	(68.7–72.8)
Ethnicity						
White	59.0	(55.2–62.9)	29.1	(26.8–31.3)	70.9	(68.7–73.2)
Black	5.9	(4.7–7.1)	34.4	(27.7–41.0)	65.6	(59.0–72.3)
Asian	19.0	(16.0–22.1)	27.9	(24.2–31.7)	72.1	(68.3–75.8)
Other	16.1	(14.6–17.6)	30.8	(27.4–34.3)	69.2	(65.7–72.6)
Parental Educational Attainment						
High School Graduate or less	12.7	(11.0–14.5)	36.2*	(31.6–40.8)	63.8	(59.2–68.4)
Some Post-Secondary Education	6.6	(5.5–7.7)	32.2*	(24.8–39.6)	67.8	(60.4–75.2)
University/College Graduate	80.6	(78.3–82.9)	28.1*	(25.9–30.2)	71.9	(69.8–74.1)
Subjective Social Status^a^						
≤4	6.1	(5.3–7.0)	35.8	(28.9–42.7)	64.3	(57.3–71.2)
≥5	93.9	(93.0–94.7)	29.0	(27.2–30.9)	71.0	(69.1–72.8)

Notes: ^a^Subjective Social Status as measured using the MacArthur Scale of Subjective Social Status (Scale from 1 to 10; mean = 7.03; standard error: 0.04); Second-order Rao-Scott Modified Chi Squared tests were conducted to examine differences between each characteristic (i.e., between likelihood of overweight/obesity in males and females) *P-value< 0.05; **P-value< 0.01

Of students between the ages of 11–17 years, 20% were overweight and 10% were obese. Males were significantly more likely to be overweight and obese compared to females (33.4% vs. 25.3%) (Table 1). Students with parents who had completed University or College had a significantly lower percentage of overweight and obesity (28%, *p*-value< 0.05) relative to those with lower levels of parental educational attainment (Table 1).

**Fig. 1:**
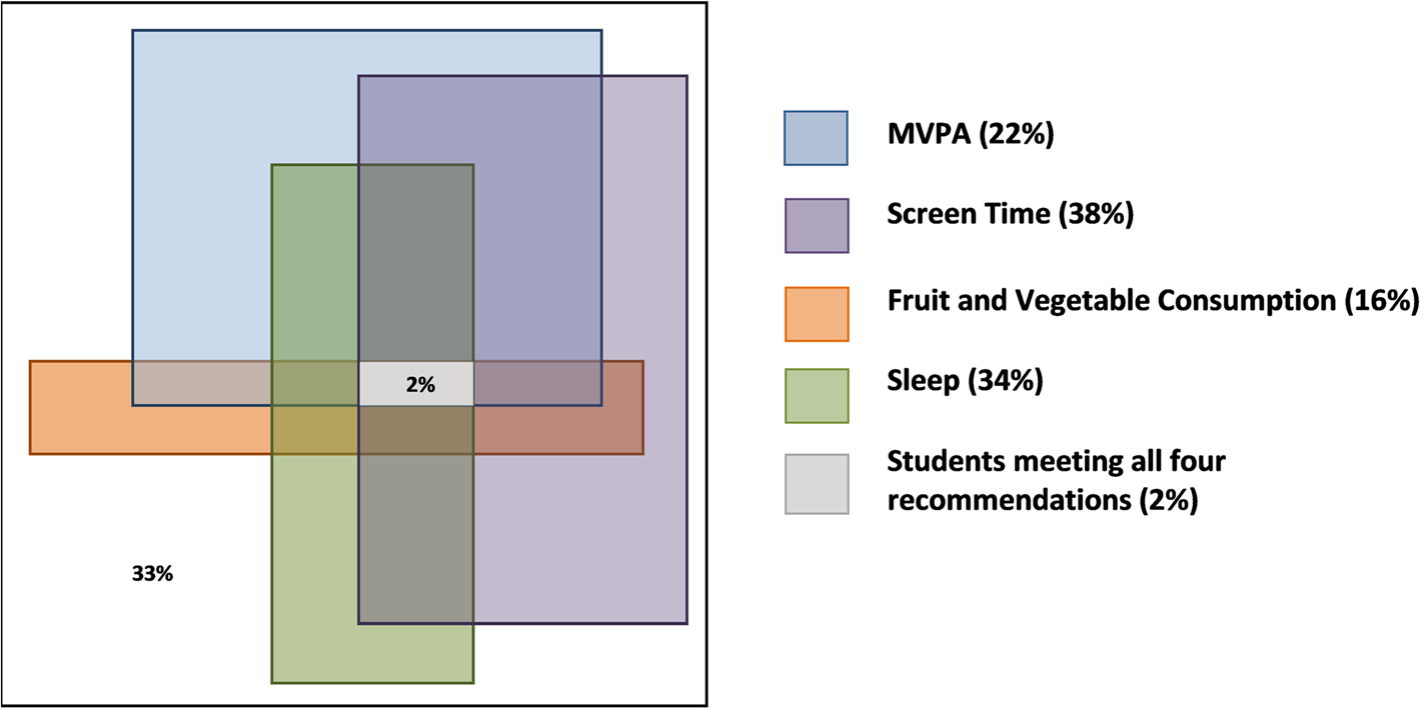
Percentage of Overall Students in Grades 7–12 Meeting Recommendations for Healthy Weight Behaviours in Ontario, 2015. Note: The size of each rectangle proportionately represents the percentage ofstudents meeting the recommendations for each behaviour; Value in white space represents percentage of students not meeting any of the four healthyweight behaviours." should be underneath the figure

More students were observed to be meeting individual healthy weight behaviour recommendations in comparison to multiple recommendations. The greatest percentage of students met the recommendations for screen time (38%); followed by 34% for sleep, 22% for MVPA, and 16% for fruit and vegetable consumption. The percentage of students meeting recommendations differed across various combinations of healthy weight behaviours (Fig. 1). One-third (33%) of students did not meet any of the four recommendations for healthy weight behaviours. Only 2% of students met the guidelines for all four behaviours (Fig. 1).

**Fig. 2:**
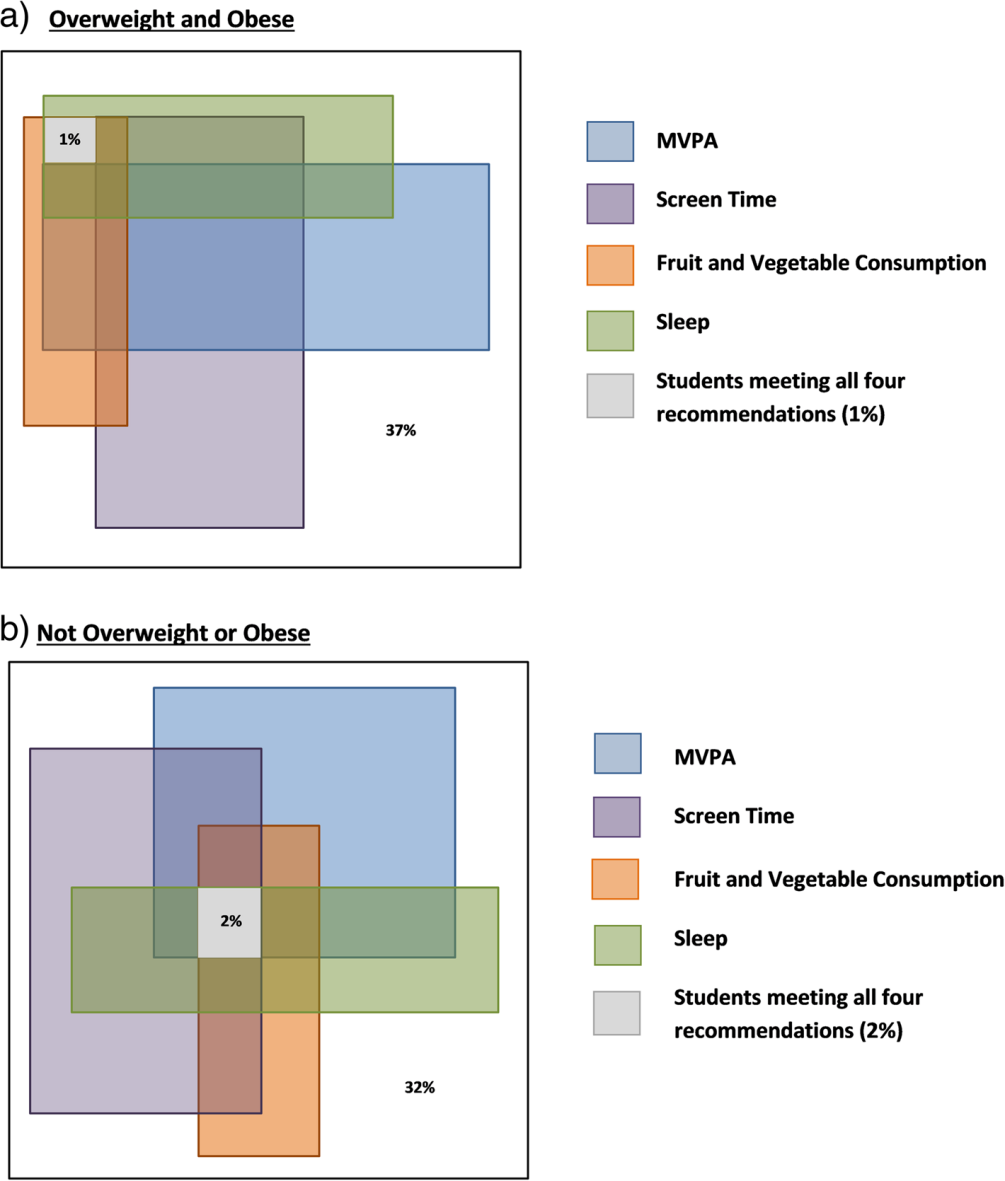
Percentage of Overweight/Obese and Not Overweight/Obese Students in Grades 7–12 Meeting Guidelines for Healthy Weight Behaviours in Ontario, 2015. **a** Overweight and Obese. **b** Not overweight and obese. Note: The size of each rectangle proportionately represents the percentage of students meeting the recommendations for each behaviour; Value in white space represents percentage of students not meeting any of the four healthy weight behaviours Note: The size of each rectangle proportionately represents the percentage of students meeting the recommendations for each behaviour; Value in white space represents percentage of students not meeting any of the four healthy weight behaviours

Further examination by BMI status showed students who did not meet the guidelines for MVPA (30% vs. 26%), screen time (31% vs. 27%), and fruit and vegetable consumption (30% vs. 26%) had higher rates of overweight and obesity compared to those who met these recommendations (Table 2). Only 1% of overweight/obese students met all four recommendations, which was not statistically significant (*p* > 0.05) from non-overweight/obese students (2%) (Fig. 2). Students not meeting any of the four recommendations were five percentage points higher among those who were overweight/obese (37%), compared to students who were not overweight/obese (32%) (p > 0.05).

**Table 2:** Bivariate association between healthy weight behaviors and BMI status

Healthy Weight Behaviours	Not Obese/Overweight	Obese/Overweight			Obese			Overweight		
	%	%	OR	95% CI	%	OR	95% CI	%	OR	95% CI
MVPA										
< 60 mins	70	30	1.24**	(1.06–1.45)	10	1.32*	(1.02–1.7)	20	1.21	(1–1.46)
≥ 60 mins	74	26	1.00		8	1.00		18	1.00	
Screen Time										
> 2 h	69	31	1.17*	(1.03–1.34)	10	1.19	(0.96–1.49)	20	1.16	(1–1.35)
≤ 2 hours	73	27	1.00		9	1.00		18	1.00	
Fruit and Vegetable Consumption										
< 5/day	70	30	1.23*	(1.04–1.45)	10	1.03	(0.79–1.35)	20	1.35**	(1.08–1.68)
≥ 5 day	74	26	1.00		10	1.00		16	1.00	
Sleep										
Inadequate^a^	70	30	1.11	(0.96–1.27)	10	1.08	(0.84–1.4)	20	1.12	(0.95–1.31)
Adequate^b^	72	28	1.00		9	1.00		19	1.00	

Notes: Reference category is not overweight or obese; OR = Odds Ratio; 95% CI = 95% Confidence Interval; ^a^Inadequate sleep = Less than 9–11 h (11–13 year olds), less than 8–10 h or more than 10 h (14–17 year olds) ^b^Adequate sleep = 9–11 h (11–13 year olds), 8–10 h (14–17 year olds); *P-value< 0.05; **P-value< 0.01

Not meeting the recommendation for MVPA showed a significant association with overweight and obesity. Students who did not meet the recommendation for MVPA had 1.24 higher odds (95% CI: 1.06–1.45) for overweight and obesity (Table 2). This difference was not significant after adjusting for the other three healthy weight behaviours (Table 3, Model 1). However, when age, sex, and social status (Model 2); parental education (Model 3); and ethnicity (Model 4) were added sequentially, MVPA showed significant differences by obesity and overweight status. After adjusting for multiple covariates, including age, sex, SSS, parental educational attainment and ethnicity, students who did not meet the recommendations for MVPA showed 1.29 higher odds (95% CI: 1.03–1.62) for overweight and obesity (Table 3, Model 4). Not meeting recommendations for sleep and fruit and vegetable consumption showed significant associations with overweight and obesity in the unadjusted model (Table 2), but the associations were not significant in the adjusted models (Table 3). Across demographics, it was observed that males showed greater odds of being overweight and obese, while students with parents with an educational attainment of High School education or less showed higher odds of being overweight and obese across all four models. Ethnicity was not a significant predictor of overweight and obesity status among students in this age demographic.

**Table 3:** Multivariable logistic regression examining the association between overweight/obesity and healthy weight behaviours with sequential adjustment for demographic characteristics

Characteristics	Model 1		Model 2		Model 3		Model 4	
	AOR	95% CI	AOR	95% CI	AOR	95% CI	AOR	95% CI
Healthy Weight Behaviours								
MVPA								
< 60 mins	1.17	(0.98–1.39)	1.22*	(1.02–1.47)	1.27*	(1.04–1.61)	1.29*	(1.03–1.62)
≥ 60 mins	1.00		1.00		1.00		1.00	
Screen Time								
> 2 h	1.14	(0.99–1.31)	1.12	(0.97–1.30)	1.16	(1.02–1.39)	1.15	(0.98–1.34)
≤ 2 hours	1.00		1.00		1.00		1.00	
Fruit and Vegetable Consumption								
< 5/day	1.16	(0.97–1.39)	1.12	(0.94–1.34)	1.07	(0.91–1.32)	1.08	(0.88–1.31)
≥ 5 day	1.00		1.00		1.00		1.00	
Sleep								
Inadequate^a^	1.07	(0.92–1.25)	1.10	(0.94–1.29)	1.08	(0.94–1.31)	1.06	(0.91–1.05)
Adequate^b^	1.00		1.00		1.00		1.00	
Demographics								
Age			0.97	(0.91–1.03)	0.99	(0.93–1.06)	0.98	(0.92–1.05)
Sex								
Male			1.57***	(1.33–1.86)	1.65***	(1.38–1.97)	1.65***	(1.38–1.97)
Female			1.00		1.00		1.00	
Subjective Social Status^c^			0.93**	(0.89–0.98)	0.94*	(0.90–0.99)	0.95*	(0.90–1.00)
Parental Educational Attainment								
University or College					1.00		1.00	
Some Post-Secondary					1.11	(0.75–1.64)	1.08	(0.73–1.60)
High School or Less					1.44*	(1.14–1.83)	1.40*	(1.11–1.78)
Ethnicity								
White							1.00	
Black							1.27	(0.89–1.82)
Asian							0.94	(0.76–1.16)
Other							1.05	(0.84–1.29)
Model Fit Statistics								
AIC	948,941.20		938,443.28		826,815.89		813,784.76	
BIC	949,848.38		938,450.34		826,822.82		813,791.66	
2 Log L	948,939.20		938,441.28		826,813.89		813,782.76	

Notes: Reference category for obese and overweight is not overweight or obese; AOR = Adjusted Odds Ratio; 95% CI = 95% Confidence Interval ^a^Inadequate sleep = Less than 9–11 h (11–13 year olds), less than 8–10 h or more than 10 h (14–17 year olds);; ^b^Adequate sleep = 9–11 h (11–13 year olds), 8–10 h (14–17 year olds); ^c^Subjective Social Status, as measured using the MacArthur Scale of Subjective Social Status (Scale from 1 to 10); *P-value< 0.05; **P-value< 0.01; ***P-value< 0.001

When these outcomes were further divided into separate overweight, obesity, and not overweight and obese statuses, not meeting the recommendations for MVPA continued to be the only behaviour significantly associated with increased odds of being obese in students within this age demographic (Table 4). Students who did not meet the recommendation for MVPA had 1.32 higher odds (95% CI: 1.02–1.7) of obesity (Table 2). After controlling for other covariates, the odds for obesity among students who did not meet the guidelines for physical activity increased to 1.45 (95% CI: 1.05–1.99) compared to students who were not overweight or obese (Table 4). Not meeting recommendations for fruit and vegetable consumption showed higher odds for overweight (OR = 1.35; 95%CI = 1.08–1.68) (Table 2), however, this was not significant in the multivariable model (Table 4). As observed previously, male students continued to show significantly higher odds of being obese, while students with parents who attended High School or less were at greater odds of being obese. Overall, these findings suggest that not meeting the recommendations for physical activity appears to be a critical behavioral factor in the presence of obesity in 11–17 year old students, even after adjustment for multiple covariates.

**Table 4:** Multinomial logistic regression examining association between healthy weight behaviours and obesity overweight status by age, sex, SSS, parental educational attainment and ethnicity

Characteristics	Obese		Overweight	
	AOR	95% CI	AOR	95% CI
Healthy weight behaviours				
MVPA				
< 60 mins	1.45*	(1.05–1.99)	1.23	(0.96–1.58)
≥ 60 mins	1.00		1.00	
Screen Time				
> 2 h	1.14	(0.88–1.47)	1.15	(0.98–1.36)
≤ 2 hours	1.00		1.00	
Fruit and vegetable consumption				
< 5/day	0.87	(0.66–1.15)	1.20	(0.93–1.56)
≥ 5 day	1.00		1.00	
Sleep				
Inadequate^a^	1.08	(0.79–1.47)	1.06	(0.90–1.25)
Adequate^b^	1.00		1.00	
Demographics				
Age	0.96	(0.87–1.06)	0.99	(0.92–1.07)
Sex				
Male	2.08***	(1.58–2.74)	1.47***	(1.20–1.82)
Female	1.00		1.00	
Subjective Social Status^c^	0.90**	(0.83–0.97)	0.97	(0.92–1.03)
Parental Educational Attainment				
High School or Less	1.93***	(1.38–2.71)	1.16	(0.91–1.50)
Some Post-Secondary	1.12	(0.69–1.83)	1.06	(0.67–1.68)
University or College	1.00		1.00	
Ethnicity				
White	1.00		1.00	
Black	1.58	(0.89–2.82)	1.12	(0.79–1.58)
Asian	0.97	(0.70–1.35)	0.92	(0.75–1.13)
Other	1.04	(0.74–1.47)	1.05	(0.83–1.32)
Model Fit Statistics				
AIC	1,066,222.90			
BIC	1,066,236.70			
2 Log L	1,066,218.90			

Notes: Reference category for obese and overweight is not overweight or obese**;** AOR = Adjusted Odds Ratio; 95% CI = 95% Confidence Interval; ^a^Inadequate sleep = Less than 9–11 h (11–13 year olds), less than 8–10 h or more than 10 h (14–17 year olds) ^b^Adequate sleep = 9–11 h (11–13 year olds), 8–10 h (14–17 year olds); ^c^Subjective Social Status, as measured using the MacArthur Scale of Subjective Social Status (Scale from 1 to 10 *P-value< 0.05; **P-value< 0.01; ***P-value< 0.001

## Discussion

This study indicated that the prevalence of students meeting multiple healthy weight recommendations was surprisingly low, and that out of all four of the behaviours examined, not meeting the recommendations for MVPA was the only significant behavioural predictor of obesity status among students within this age demographic.

There is a limited body of research examining the simultaneous role of MVPA, screen time, diet and sleep on adolescent weight status, and lacking consistency in the measurement of these behaviours at the international level [13–15]. To the authors’ knowledge, this is the first study to examine the prevalence of students meeting the recommendations for these four healthy weight behaviours in a Canadian context. Consequently, the observed prevalence rates of students meeting healthy weight behaviour recommendations could not be compared with other reported Canadian findings, as no other study has examined all four of these rates at the national level. However, existing studies have evaluated the combined impact of three out of the four behaviours addressed in the current study [11, 27–34]. Notably, Roman-Vinas et al. investigated the global prevalence of 9–11 year olds meeting the recommendations for MVPA, screen time and sleep, and observed that only 7% of children worldwide are adhering to all three, which is comparable to the results observed in the current study (4%, not shown in Fig. 1) [28]. Carson et al. also explored the impact of these three behaviours among 6–17 year-old Canadian children and adolescents, observing 17% of participants to be complying with the same three guidelines [11]. While our results are most comparable to the global prevalence of children meeting recommendations for MVPA, screen time and sleep, differences between our findings and other Canadian research is striking. However, these discrepancies could be attributed to differences in study population age (ie. Roman-Vinas et al., 2016 and Carson et al., 2017 included participants younger than eleven years of age), and methodology and measurement, such as self-report vs. objective/accelerometry-based measures for MVPA, sleep duration, and anthropometry measurements [11, 28].

In this study of adolescents, not meeting recommendations for MVPA appeared to have the strongest association for obesity, when compared with other lifestyle behaviours. The isolated relationship between MVPA and healthy weight status has been well-documented in the literature, attributable to the direct physiological impact of adequate physical activity on adiposity, which promotes the establishment of an energy balance between consumed and expended calories [35–37]. Existing studies have presented findings comparable with the results from this study [28–30, 38]. Wilkie et al. studied possible associations between not meeting healthy weight recommendations for all four healthy weight behaviours and BMI z-score in a UK-based sample of adolescents between the ages of 9–11, noting that participants who did not meet the recommendations for physical activity had a significantly higher BMI z-score compared to those who were classified as adequately active [14]. The study by Roman-Vinas et al., which evaluated a sample that included Canadian participants, noted that 9–11 year olds that did not meet the MVPA guideline showed the highest odds for obesity, when compared to not meeting guidelines for screen time and sleep [28]. Laurson et al. also found an analogous relationship between MVPA and obesity, observing that not meeting the MVPA guideline was the only behaviour that resulted in an increased likelihood of being obese in a sample of American adolescents in grades 9–12, regardless of whether screen time or sleep guidelines were adhered to [29].They also found that adolescents not meeting the recommendations for physical activity were at an increased odds of not meeting (or exceeding) the recommendations for screen time [29, 38]. This finding presents a possible explanation for the observed association in this study between not meeting the recommendation for MVPA and obesity status in our study, as adolescents may be using time that could otherwise be spent being physically active to engage in behaviours with negative impacts on weight status, such as excessive screen time and consumption of sugar-sweetened foods and beverages. While there is considerable evidence to suggest MVPA as being the strongest behavioural determinant of adiposity, these findings support the notion that lifestyle behaviours synergistically contribute to BMI status, and reinforce the need to study the relationships between these behaviours and weight status concurrently [39, 40].

Our findings also indicated a number of socio-demographic factors to be significantly predictive of obesity status in adolescents between the ages of 11–17 years. Males had increased odds of being obese in comparison to females within this age demographic, a tendency in North-American adolescents that has been previously acknowledged within the literature [4, 41, 42]. Differences in gender-specific biology, genetics, and behaviours such as self-reporting are possible sources for these discrepancies, and are further compounded by subjective cultural, environmental and socio-economic circumstances [42]. Findings from our study also showed adolescents to be at greater odds of being obese based on lower SSS ratings and lower parental education levels. A study exploring the associations between SSS ratings and overweight and obesity noted similar results, demonstrating the potential impacts of subjective perceptions of society on health outcomes among school-aged adolescents [24]. Our finding that lower parental education levels (used as a proxy for socio-economic status (SES)) were significantly predictive of greater odds of obesity was also consistent with previous research conducted among US and Canadian children and adolescents between the ages of 10–17 years, which have identified an inverse relationship between household SES and obesity in higher economic status countries [43, 44]. In examining the associations between socio-demographic factors and obesity among adolescents, there currently exists a literature gap in the body of research that has examined this relationship in conjunction with healthy weight behaviours. Our research is one of few to have not only examined socio-demographic characteristics as individual covariates, but to have addressed the role of sex, child’s perceived social status and parental educational attainment on obesity status in a study primarily focused on the interactive impact of multiple healthy weight behaviours. While this level of analysis may facilitate a more comprehensive understanding of the complexities in the behavioural and social interactions affecting adolescent overweight and obesity, further research examining the joint effects of lifestyle and environmental factors is necessary in order to address the true role of social disparities on weight status among adolescents [43].

### Limitations

Several limitations should be taken into consideration when interpreting the findings of this study. The cross-sectional nature of this study prevents us from making any causal or reverse-causal inferences from the study results, as the temporality of overweight or obese weight status cannot be established. All responses in OSDUHS are self-reported, leading to potential recall and social desirability bias [16]. A study comparing the differences in self-reported vs. direct height and weight measurements in Canadians noted self-reported height to be typically greater than measured height, while self-reported weight was lower than measured weight [45]; consequently, self-reported rates of overweight and obesity were lower than rates provided by direct measures. This suggests that the rates provided in this study may underestimate the true magnitude of overweight and obesity in Ontario’s adolescents. Differences in self-reporting by sex have generally been minimal, though a few studies have shown a greater tendency for misreporting height and weight in overweight and obese female adolescents [45–49]. Studies examining the validity of self-reported health behaviour measures have noted that adolescents commonly over-report their engagement in the study’s healthy weight behaviours [50–53]. While self-report surveys function as a useful tool for the purposes of behavioural trend surveillance, direct measures for capturing these behaviours would allow for more accurate measurement of compliance to Canadian recommendations [50–53]. In addition, the self-report measure used for fruit and vegetable consumption determined fruit and vegetable consumption based on frequency of daily consumption, and not relative to servings consumed. Therefore, phrasing of the survey question, in addition to the range of response categories offered to study participants in the survey, inhibited any comparisons to Health Canada’s *Canada Food Guide* guidelines. However, the cut-off for fruit and vegetable consumption used in this study has been used in previous literature, and is comparable to thresholds used world-wide [20, 21, 54]. Findings from this study cannot be applied to Ontario students who were excluded from the 2015 OSDUHS sample, including students enrolled in private schools, First Nations schools on reserve, military bases, in the remote northern region of Ontario and those in geographically inaccessible areas [16]. All students excluded from the OSDUHS sample collectively represent approximately 8% of the Ontario student population in grades 7–12.

## Conclusions

This study highlights the importance of exploring overweight and obesity across multiple lifestyle behaviours and socio-demographic characteristics, in order to accurately account for the collective influence of these factors on adolescent weight status. According to our findings, one-third of Ontario adolescents aged 11–17 years are not meeting any of the four examined healthy weight behaviour recommendations. The implications of this alarming statistic should be considered promptly by stakeholders and decision-makers developing programs and policies to improve health outcomes among Ontarians within this age bracket. The finding that fruit and vegetable consumption was the recommendation least complied with by adolescents also indicates that more research inclusive of this behaviour, alongside other healthy lifestyle behaviours, is warranted. Our study also noted that MVPA was consistently the sole predictor of overweight and obesity status among these four behaviours, suggesting that this behaviour may be the most important behavioural target for interventions aiming to prevent and manage obesity in adolescents. Further research examining the combined impact of these lifestyle behaviours on overweight and obesity status is necessary in order to comprehensively address this public health issue.

## Data Availability

The data that support the findings of this study are available from the Centre for Addiction and Mental Health but restrictions apply to the availability of these data, which were used under license for the current study, and so are not publicly available. Data are however available from the authors upon reasonable request and with permission of CAMH. The 2015 OSDUHS data is stored at the Centre for Addiction and Mental Health (CAMH) as well as Public Health Ontario. For access, please contact the corresponding author.

## Abbreviations

AOR: Adjusted odds ratio
APHEO: Association of Public Health Epidemiologists of Ontario
BMI: Body mass index
CI: Confidence interval
MVPA: Moderate-to-vigorous physical activity
OSDUHS: Ontario student drug-use and health survey
SD: Standard deviation
SSS: Subjective social status
WHO: World Health Organization
